# 14-3-3ζ and aPKC-ι synergistically facilitate epithelial-mesenchymal transition of cholangiocarcinoma via GSK-3β/snail signaling pathway

**DOI:** 10.18632/oncotarget.10483

**Published:** 2016-07-08

**Authors:** Yan Yang, Yan Liu, Jun-chuang He, Jian-ming Wang, Peter Schemmer, Chao-qun Ma, Ya-wei Qian, Wei Yao, Jian Zhang, Wei-peng Qi, Yang Fu, Wei Feng, Tao Yang

**Affiliations:** ^1^ Department of Biliary and Pancreatic Surgery/Cancer Research Center Affiliated Tongji Hospital, Tongji Medical College, Huazhong University of Science and Technology, Wuhan, Hubei 430030, China; ^2^ Department of General and Transplant Surgery, University Hospital Heidelberg, Heidelberg 69120, Germany

**Keywords:** cholangiocarcinoma, epithelial-mesenchymal transition, synergy, transfection, silencing

## Abstract

Cholangiocarcinoma (CCA) invasion and metastasis are the primary causes of poor survival rates in patients. The epithelial-mesenchymal transition (EMT) is a crucial step in cancer invasion and metastasis. However, it is still unclear of the molecular mechanism. In this study, the expression of 14-3-3ζ and atypical protein kinase C-ι (aPKC-ι) was further detected in CCA tissues and cell lines. Meanwhile, we established the EMT model of CCA cells and investigated 14-3-3ζ and aPKC-ι co-regulatory effect on the EMT *in vitro* and *in vivo*. Further, we identified the downstream molecular glycogen synthase kinase 3 beta (GSK-3β)/Snail signalling pathway that contribute to regulating the EMT. Our data showed that the expression of 14-3-3ζ and aPKC-ι was synergistically increased in CCA tissues compared with adjacent noncancerous tissues and was intimately associated with differentiation and the tumour-node-metastasis (TNM) stage. Multivariate Cox regression analysis indicated that high 14-3-3ζ and aPKC-ι expression separately predicted a poor prognosis and were independent prognostic indicators in patients with CCA. The CO-IP experiment confirmed that the mutual binding relationship between 14-3-3ζ and aPKC-ι. Small interfering RNAs and siRNA rescue experiment demonstrated that 14-3-3ζ and aPKC-ι regulated each other. In addition, 14-3-3ζ and aPKC-ι pretreatment by si-RNA inhibit the phosphorylated GSK-3β and Snail expression during EMT. Meanwhile, silence of 14-3-3ζ or aPKC-ι suppressed CCA cells migration, metastasis and proliferation *in vitro* and *in vivo*. Our study demonstrates that 14-3-3ζ and aPKC-ι synergistically facilitate EMT of CCA via GSK-3β/Snail signalling pathway, and may be potential therapeutic target for CCA.

## INTRODUCTION

Cholangiocarcinoma (CCA) is an epithelial cell malignancy deriving from the intrahepatic and extrahepatic biliary trees [1]. The incidence of CCA has been increasing annually in China [2], however, most CCA patients are notoriously difficult to diagnose and have poor prognosis [1, 3]. Currently, radical resection is the only effective treatment, with the 5-year survival rate after R0 resection ranging from 20% to 40%[4] and the rate of postoperative recurrence up to 75%[5]. The reason is that CCA cells possess a strong propensity to invade and metastasize to adjacent organs. Unfortunately, the molecular mechanisms underlying invasion and metastasis remain elusive.

Epithelial carcinoma cells shed themselves of cell-cell junctions and migrate to a distal site where they recolonize via epithelial-mesenchymal transition (EMT) [6]. The EMT has been shown to be a pivotal mechanism contributing to cancer invasion and metastasis [7]. During the processes of EMT, epithelial cells lose their polarity and acquire the migratory properties of mesenchymal cells [8], including the down-regulating of E-cadherin and β-catenin, and the up-regulation of N-cadherin and vimentin. Transcription factors, such as Snail, Slug, ZEB1 and Twist, have been implicated in the control of EMT. Snail is a zinc finger transcription factor which has been established as a key EMT regulator [9]. Snail and Glycogen synthase kinase 3 beta (GSK-3β) together function as a molecular switch for many signaling pathways that strongly repress E-cadherin expression [10].

Meanwhile, EMT is mediated by a complex network of signaling pathways. In this network, atypical protein kinase C-iota (aPKC-ι), one of polarization regulatory proteins, acts as a central hub [11]. It is well-known that aPKC-ι plays an essential role in controlling the organization of E-cadherin in different cell types [12, 13]. Our previous studies also showed that the synergistic expression of aPKC-ι and E-cadherin may reflect the differentiation and invasive potential of CCA and aPKC-ι may play an important role in inducing EMT [14]. A recent study found that aPKC-ι can directly phosphorylate GSK-3β and lead to defects in apical-basal polarity [15]. Interesting, the activity of GSK-3β is mainly regulated by its Ser9 phosphorylation, and 14-3-3ζ enhances Ser9 phosphorylation of GSK-3β by PKC [16].

14-3-3ζ has been proposed to be directly involved in cellular transformation and proliferation [17], and associated with prognosis of cancers [18]. A striking feature of 14-3-3 proteins is that they are able to bind to many signaling molecules and have therefore been implicated in a wide array of cellular activities, including cell proliferation, migration, and the EMT [19]. Studies have indicated that 14-3-3ζ is overexpressed in CCA [20] and that up-regulation of 14-3-3ζ contributes to resistance of tumor cells to apoptosis and increases the potential for invasion and metastasis by facilitating EMT [21–24]. Therefore, 14-3-3ζ has the potential to serve as a molecular target for cancer therapy [25]. However, the mutual relationship between 14-3-3ζ and aPKC-ι in CCA are unclear. On the basis of previous works, we further detected the expression of 14-3-3ζ and aPKC-ι in CCA tissues, examined the interaction between them in CCA cells, investigated the regulation of EMT by them *in vitro* and *in vivo*, with an attempt to explore the mechanism of EMT.

## RESULTS

### Co-overexpression of 14-3-3ζ and aPKC-ι promotes tumor progression and predicts poor prognosis in CCA patients

**Figure 1:**
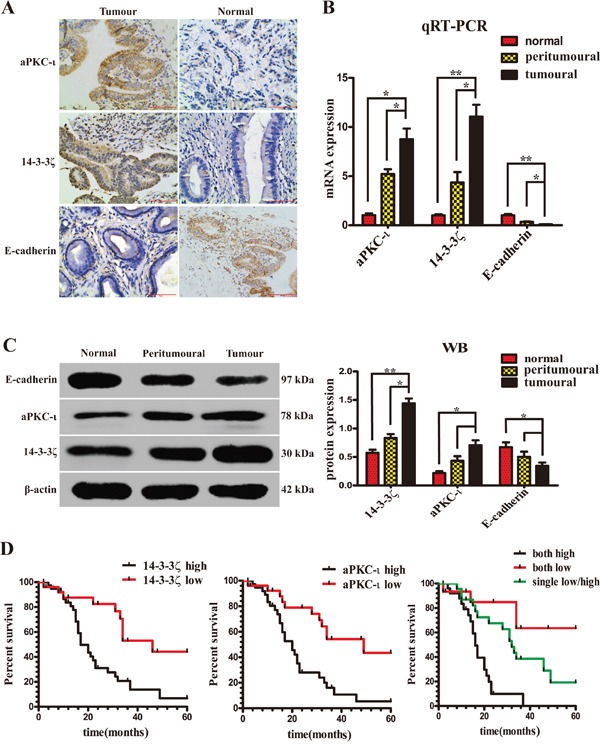
Expression of 14-3-3ζ, aPKC-ι and E-cadherin in patients with CCA. The expression of 14-3-3ζ and aPKC-ι correlates with the prognosis of patients **A.** Representative IHC staining of the expression of aPKC-ι, 14-3-3ζ and E-cadherin protein in CCA tissue (left panels) and normal tissue (right panels) were shown (400×). Scale bar, 100μm. **B.** The mRNA expression of 14-3-3ζ, aPKC-ι and E-cadherin in tumor, peritumor and normal tissues were determined by qRT-PCR. β-actin was used as the loading control. The data were shown as mean±SD. Fold changes were calculated through relative quantification (2^−ΔΔCt^). The total RNA was isolated from at least three samples of each tissue. **, *P*<0.001 and *, *P*<0.05. **C.** The protein expression of 14-3-3ζ, aPKC-ι and E-cadherin were determined by Western Blot. β-actin was used as the loading control. All of the experiment were repeated 3 times and yielded similar results. Representative image was shown (left panel). Statistical analysis of relative optical density of each band was shown(right panel). **, *P*<0.001 and *, *P*<0.05. **D.** Kaplan-Meier analysis. left panel: Patients with low 14-3-3ζ group (n=26) experienced a significantly longer overall survival (OS) than patients with high 14-3-3ζ group (n=38) (median OS: 46 months *vs* 17 months, *P*=0.001, log-rank test); middle panel: Patients with low aPKC-ι group (n=26) experienced a significantly longer OS than patients with high aPKC-ι group (n=38) (median OS: 49 months *vs* 20 months, *P*<0.001, log-rank test); right panel: Patients with co-expression low 14-3-3ζ and aPKC-ι group (n=14) experienced a significantly longer OS than patients with co-expression high 14-3-3ζ and aPKC-ι group (n=27), or only single expression low 14-3-3ζ or aPKC-ι group (n=23) (median OS: 49.8 months *vs* 17 months *vs* 34.5 months, *P*<0.001, log-rank test).

Initially, the expression of 14-3-3ζ, aPKC-ι and E-cadherin in 64 paired CCA and peritumoral tissue samples and 10 choledochocyst samples as controls was detected using immunohistochemistry (IHC), quantitative real-time polymerase chain reaction (qRT-PCR) and western blotting (WB), meanwhile the patients’ samples were histopathologically evaluated (Table 1). The average expression of 14-3-3ζ and aPKC-ι was significantly higher in CCA samples than in benign bile duct tissues. In contrast, the expression of E-cadherin was significantly lower (*P*<0.05; Figure 1A, 1B & 1C). In IHC experiment, high expression (score >4) or low expression (score ≤4) of 14-3-3ζ was detected in 60.9% (39/64) *vs.* 39.1% (25/64) of CCA tissues, 25% (16/64) *vs.* 75% (48/64) of peritumoral tissues (χ^2^=16.865, *P*<0.001), 10% (1/10) *vs.* 90% (9/10) of choledochocyst tissues (χ^2^=9.035, *P*=0.003). Similarly, high expression or low expression of aPKC-ι was detected in 59.4% (38/64) *vs.* 40.6% (26/64) of CCA tissues, 23.4% (15/64) *vs.* 76.6% (49/64) of peritumoral tissues (χ^2^=17.034, *P*<0.001), 0% (0/10) *vs.* 100% (10/10) of choledochocyst tissues (χ^2^=12.205, *P*<0.001). On the contrary, as a key marker of EMT, E-cadherin expression was significantly lower in CCAs than in peritumoral or choledochocyst tissues (*P*<0.05; Figure 1A, 1B & 1C).

**Table 1:** Co-expression of aPKC-ι and 14-3-3ζ in different clinical TNM stage and pathological differentiation of cholangiocarcinoma

Group	N	aPKC-ι		*P*	14-3-3ζ		*P*
		Low	High		Low	High	
Cancerou/noncancerou tissues:							
Cholangiocarcinoma	64	31	33	0.002	28	36	0.007
Choledochocyst	10	10	0		9	1	
Carcinoma/pericarcinoma tissues:							
Carcinoma	64	31	33	0.001	28	36	<0.001
Pericarcinoma	64	49	15		48	16	
Clinical TNM stage:							
I-II	41	27	14	<0.001	24	17	0.001
III-IV	23	4	19		4	19	
Differentiation:							
Well	23	17	6	0.002	16	7	0.002
Medium/Poorly	41	14	27		12	29	

Moreover, over-expression of 14-3-3ζ in patients at TNM stages I-II was 41.5% (17/41), and at TNM stages III-IV was 82.6% (19/23) (χ^2^ =10.136, *P*=0.001). And over-expression of aPKC-ι in patients at TNM stages I-II was 34.1% (14/41), and at TNM stages III-IV was 82.6% (19/23) (χ^2^ =13.856, *P*<0.001). Over-expression of 14-3-3ζ in patients with well differentiation was 30.4% (7/23), and with medium/poorly differentiation was 70.7% (29/41) (χ^2^=9.722, *P*=0.002). And over-expression of aPKC-ι in patients with well differentiation was 26.1% (6/23), and with medium/poorly differentiation was 65.9% (27/41) (χ^2^ =9.329, *P*=0.002)(Table 1). 14-3-3ζ and aPKC-ι remained a significant risk factor in patients at TNM stages III-IV with moderate and poor differentiation compared with those at TNM stages with I-II with good differentiation. Further there is a significant positive correlation between these two proteins (γ=0.406, *P*=0.001). Kaplan-Meier analysis showed that patients with high-expression of 14-3-3ζ or aPKC-ι had shorter overall survival as compared to those with low-expression of 14-3-3ζ or aPKC-ι (14-3-3ζ: χ^2^ =10.140, *P*= 0.001; aPKC-ι: χ^2^ =12.575, *P*<0.001, respectively) (Figure 1D). Multivariate COX regression analysis showed that both 14-3-3ζ and aPKC-ι were independent factors on the survival prognosis of patients with CCA (14-3-3ζ: *P* =0.026; aPKC-ι: *P* =0.025, respectively) (Table 2).

**Table 2:** Univariate and multivariate analysis of factors associated with survival of 64 CCA patients

	Survival		
	univariate analysis	multivariate analysis	
	*P* value	95%Cl	*P* value
Age(>60 vs ≤60)	0.742		
Gender(male vs female)	0.700		
TNM stage(I-II vs III-IV)	0.001	0.069-0.671	0.008
Tumor differentiation(well vs medium/poorly)	<0.001	0.095-0.565	0.001
aPKC-ι expression(low vs high)	<0.001	0.151-0.879	0.025
14-3-3ζ expression(low vs high)	<0.001	0.184-0.897	0.026

Taken together, these data indicated that the expression of 14-3-3ζ and aPKC-ι was synergistically elevated in contrast to repressed E-cadherin expression in CCA. Overexpression of 14-3-3ζ and aPKC-ι were predictors of poor prognosis in patients and may synergistically contribute to the progression of disease through regulation EMT procedure.

### 14-3-3ζ binds to aPKC-ι in CCA tissues and cells

**Figure 2:**
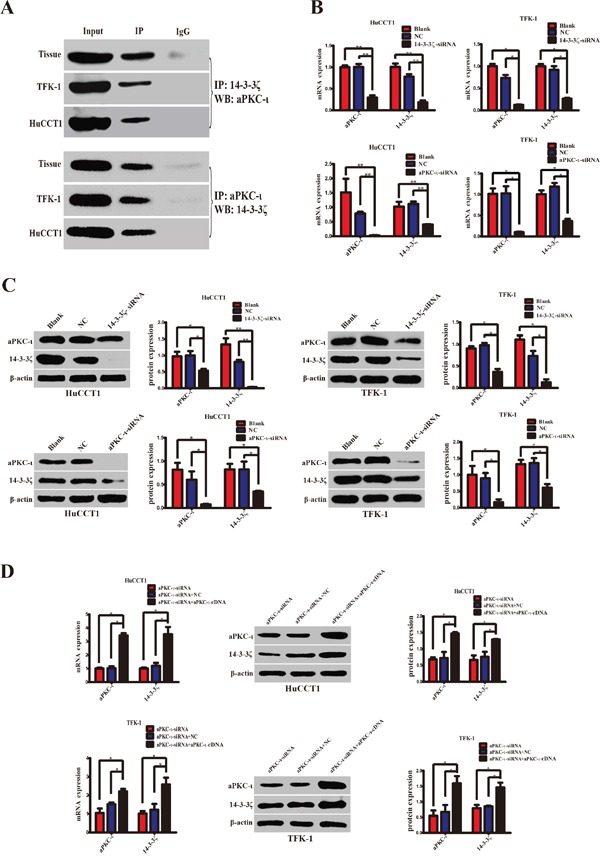
14-3-3ζ cooperates with aPKC-ι and regulates mutually **A.** Specific protein-protein interactions between 14-3-3ζ and aPKC-ι in CCA tissues and cell lines (TFK-1 and HuCCT1) were determined by CO-IP. The supernatants of the lysates without any antibody, named Input, were used as the positive control, and proteins precipitated by IgG were used as the negative control. **B.** qRT-PCR was used to analyze the expression of 14-3-3ζ or aPKC-ι at the mRNA level in HuCCT1 (left panels) and TFK-1 (right panels) cells after transfection with siRNA lentivirus. **C.** Western Blot was used to explore the protein expression of 14-3-3ζ or aPKC-ι in the HuCCT1 and TFK-1 cells after transfection with the siRNA lentivirus. Representative image was shown (left panels). Statistical analysis of the relative optical density of each band was shown (right panels). **D.** The aPKC-ι-siRNA rescue experiment was used to inspect the expression changes of 14-3-3ζ and aPKC-ι, as indicated. Representative image was shown (left panels). Statistical analysis of the relative optical density of each band was shown (right panels). Cells that were not transfected with virus were used as the blank control, and cells that were transfected with an empty vector were used as the negative control (NC). β-actin was used as the loading control. **, *P*<0.001 and *, *P*<0.05.

Given the synergistic expression of 14-3-3ζ and aPKC-ι in CCA tissues, we performed co-immunoprecipitation (CO-IP) experiments to further explore their relationship. The total proteins were extracted from CCA tissues and cell lines (TFK-1 & HuCCT1) and were precipitated with anti-aPKC-ι antibody (Cell Signaling Technology, MA, USA) or anti-14-3-3ζ antibody (Santa Cruz Biotechnology, Santa Cruz, CA, USA). The protein precipitates were analyzed by WB with anti-14-3-3ζ antibody or anti-aPKC-ι antibody. Similar to the positive control, 14-3-3ζ or aPKC-ι was correspondingly detected in the precipitated protein complex. Conversely, the expression of 14-3-3ζ and aPKC-ι was absent in the negative control (Figure 2A). These findings revealed that 14-3-3ζ may act in combination with aPKC-ι in CCA.

### 14-3-3ζ and aPKC-ι regulates each other

**Figure 3:**
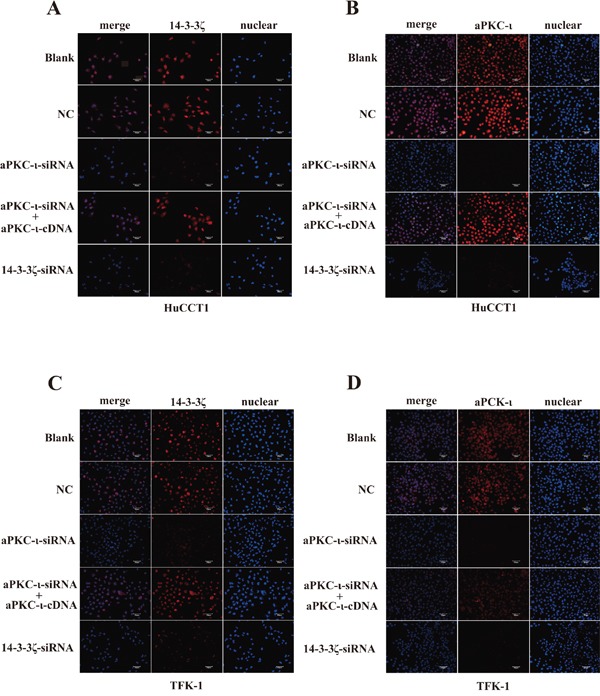
Immunofluorescence staining of 14-3-3ζ and aPKC-ι Immunofluorescence was used to detect 14-3-3ζ (red) **A & C.** and aPKC-ι (red) **B & D.** in HuCCT1 (A & B) and TFK-1 (C & D) treated with small interfering RNAs as indicated. Cells untransfected with virus were used as the blank control, and transfected with an empty vector were used as the negative control (NC). DAPI (blue) was used to stain the nuclei. The fluorescence intensity of 14-3-3ζ and aPKC-ι were stronger in both blank and NC groups, and were weaker in cells transfected with siRNA. But in the aPKC-ι-siRNA rescue experiment, the fluorescence intensity were recovered. Scale bar, 20μm. Original magnification 40×. One representative experiment out of the three performed was shown.

In view of 14-3-3ζ binds to aPKC-ι, we further examined the mutual regulatory effects of these two proteins in CCA cell lines. We stably knocked down 14-3-3ζ or aPKC-ι in TFK-1 and HuCCT1 by transfecting lentivirus containing 14-3-3ζ-siRNA or aPKC-ι-siRNA. qRT-PCR, WB and immunofluorescence (IF) showed that both 14-3-3ζ and aPKC-ι expression were decreased in the cells with small interfering RNA (siRNA) (*P*<0.05; Figure 2B & 2C and Figure 3). Subsequently, in the aPKC-ι-siRNA rescue experiment, lentiviruses carrying aPKC-ι-cDNA were re-transfected into the cells with aPKC-ι-siRNA. In addition to aPKC-ι expression being reversed, the expression of 14-3-3ζ was significantly elevated (*P*<0.05; Figure 2D and Figure 3). Collectively, these results suggested that 14-3-3ζ and aPKC-ι regulate each other.

### Knockdown 14-3-3ζ or aPKC-ι inhibits the EMT of CCA cells *in vitro*

**Figure 4:**
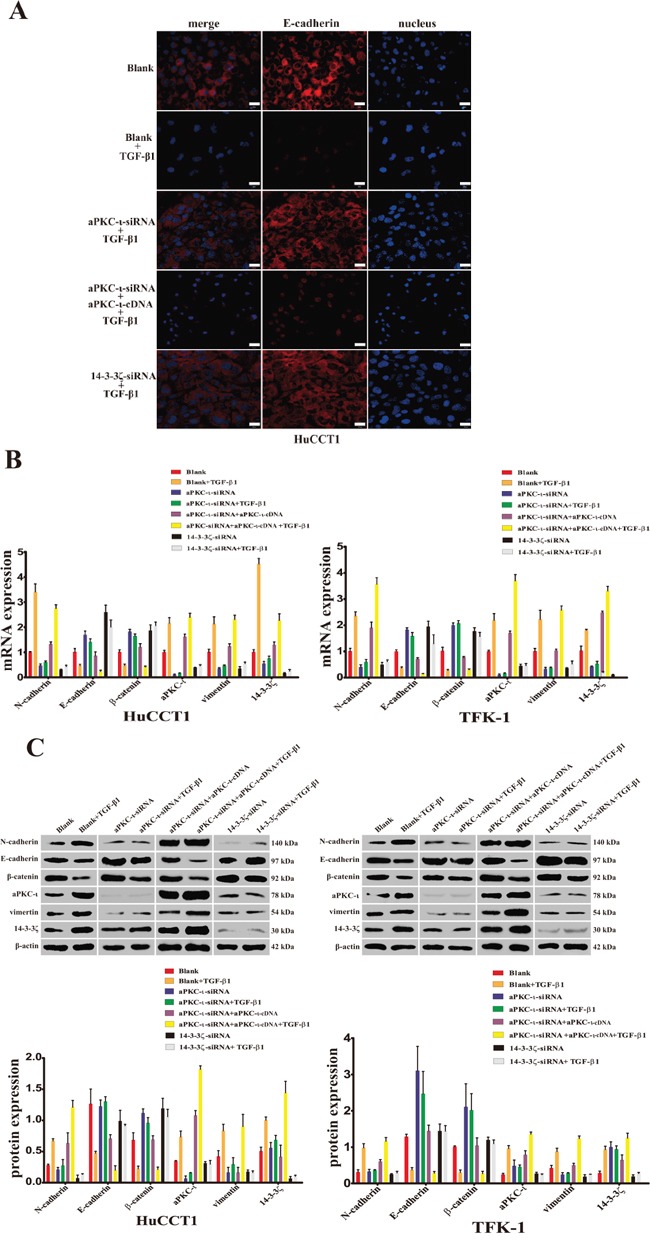
Reduced expression of aPKC-ι and 14-3-3ζ inhibits the TGF-β1-induced EMT in CCA cells *in vitro* **A.** Immunofluorescence was used to detected the fluorescence intensity of E-cadherin (red) in HuCCT1 cells treated with small interfering RNAs and cultured with TGF-β1 for 5 days as indicated. DAPI (blue) was used to stain the nuclei in the right panels, and the merged images are shown in the left panels. Cells without any treatment were used as the blank control. Scale bar, 20 μm. Original magnification 40×. One representative experiment out of the three performed was shown. **B.** qRT-PCR was used to detect the mRNA expression of 14-3-3ζ, aPKC-ι, and EMT markers (E-cadherin, N-cadherin, β-catenin and vimentin) in various cells treated with small interfering RNAs and cultured with TGF-β1 for 5 days as indicated (HuCCT1, left panel; TFKE-1, right panel). Untreated cells were used as the blank control, and β-actin was used as the loading control. **, *P*<0.001 and *, *P*<0.05. **C.** Western Blot was used to analyze the protein level expression of 14-3-3ζ, aPKC-ι, and EMT markers (E-cadherin, N-cadherin, β-catenin and vimentin) in various cells treated with small interfering RNAs and cultured with TGF-β1 for 5 days as indicated (HuCCT1, left panel; TFK-1, right panel). Representative image was captured (upper panel). Statistical analysis of the relative optical density of each band was shown (lower panel). β-actin was used as the loading control. **, *P*<0.001 and *, *P*<0.05.

Our previous studies showed that aPKC-ι plays an important role in EMT of CCA [14]. To further investigate the molecular mechanism in this process, we established the TGF-β1-induced EMT model *in vitro*. Followed knockdown 14-3-3ζ or aPKC-ι, the expression of EMT markers (including: N-cadherin, E-cadherin, β-catenin and vimentin) were detected in HuCCT1 and TFK-1 through IF, WB and qRT-PCR (Figure 4). When EMT was induced by TGF-β1, the expression of epithelial markers (E-cadherin & β-catenin) were decreased, in contrast to mesenchymal markers (N-cadherin & vimentin) increased (*P*<0.05). However, followed 14-3-3ζ-siRNA or aPKC-ι-siRNA were transfected into the CCA cells, these markers did not show distinct changes in expression in response to TGF-β1-induced EMT (*P*>0.05). Furthermore, the aforementioned changes in EMT markers were recovered in the aPKC-ι-siRNA rescue experiment (*P*<0.05). All of these findings suggest that knockdown 14-3-3ζ or aPKC-ι inhibited TGF-β1-induced EMT of CCA cells *in vitro*.

### Down-regulation of 14-3-3ζ suppress transformation, invasion, migration and proliferation of CCA cells *in vitro*

**Figure 5:**
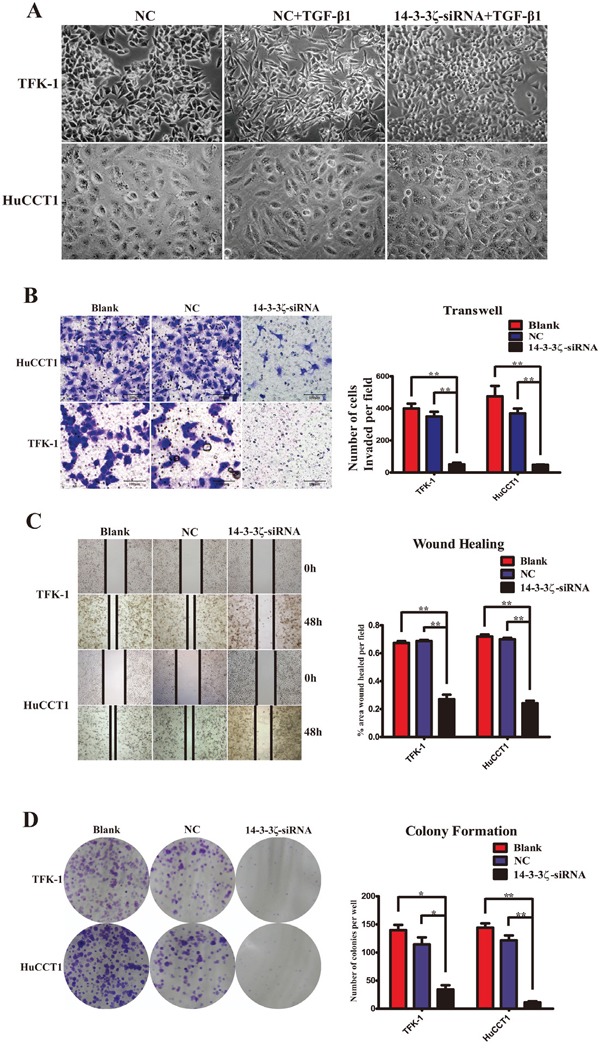
Inhibition of 14-3-3ζ repress EMT, invasion, migration and proliferation of CCA cells *in vitro* **A.** Morphological changes in cells treated with TGF-β1 (10 ng/ml) were observed under a phase-contrast microscope within 5 days (TFK-1, upper panel; HuCCT1, lower panel). Cells that were not transfected with 14-3-3ζ-siRNA were used as the negative control (NC, left panel, 400×), and those treated with TGF-β1 exhibited transition from a polarized, epithelial morphology to a depolarized, spindle-shaped and mesenchymal morphology (middle panel, 400×). However, no significant morphological changes were observed in cells with 14-3-3ζ-siRNA treated with TGF-β1 (right panel, 400×). **B.** Transwell assay was used to investigate the invasive capability of various cells, as indicated. The number of cells that invaded through the membrane was determined under a light microscope (magnification 40×; HuCCT1, upper panels; TFK-1, lower panels). Representative image was captured (left panel). Statistical analysis was shown (right panel). **C.** Wound-healing assay was employed to examine the migration capability of various cells as indicated, and solid lines represent the wound edges. Images were captured using light microscopy (magnification 4×). The migration index was calculated as described in the Materials and Methods (HuCCT1, lower panels; TFK-1, upper panels). Representative image was captured (left panel). Statistical analysis was shown (right panel). **D.** Colony-formation assays were performed to evaluate the proliferative capability of various cells as indicated (HuCCT1, lower panels; TFK-1, upper panels). Representative image was shown (left panel). Statistical comparison of the indicated groups was performed (right panel). Cells that were not transfected with virus were used as the blank control(Blank), and those transfected with an empty vector were used as the negative control (NC). **, *P*<0.001 and *, *P*<0.05. The data are represented as the mean±SD (n=3).

Considering that 14-3-3ζ regulates EMT in CCA cells, we cytologically studied the effect of 14-3-3ζ on the behavior of CCA cells. Morphologically, the transformation phenotypes of TFK-1 and HuCCT1, which cultivated by TGF-β1, were microscopically observed as a change from the short shuttle- or round-shaped epithelioid cells to fibroblasts-like mesenchymal cells. However, the number of fibroblasts-like mesenchymal cells was significantly less in those cells transfected with 14-3-3ζ-siRNA (Figure 5A). Transwell invasion assays were employed to assess cell invasion, and the data demonstrated that silencing of 14-3-3ζ expression significantly reduced the invasive abilities of the CCA cells. Fewer invading cells that adhered to the lower chamber surface were observed in the 14-3-3ζ-siRNA-treated cells compared with the blank and negative control cells (HuCCT1: 48.3±3.1 *vs.* 475.3±112.0 & 369.0±52.6, *P*=0.003 & <0.001; TFK-1: 51.0±16.8 *vs.* 399.7±52.2 & 349.0±51.2, *P*<0.001 & =0.001, respectively; Figure 5B). The wound-healing assay was performed to assess cell migration, and the findings indicated that healing occurred more slowly with the cells transfected with 14-3-3ζ-siRNA than with the blank or negative control cells at 48 h (TFK-1: *P*<0.001; HuCCT1: *P*<0.001; Figure 5C). A colony-formation assay was used to investigate cell proliferation, and the results showed that the number of colonies formed was significantly decreased in the cells in which 14-3-3ζ was down-regulated compared with the blank or negative controls (TFK-1: 34.0±13.5 *vs.* 139.7±16.2 & 114.3±21.7 cells per well, *P*=0.001 & =0.006, respectively; HuCCT1: 11.0±4.0 *vs.* 144.0 ±13.2 & 121.7±15.0 cells per well, *P*<0.001 & <0.001, respectively, Figure 5D). These findings suggested that inhibition of 14-3-3ζ using siRNA can suppress transformation, invasion, migration and proliferation of CCA cells *in vitro*.

### Silence of 14-3-3ζ repress CCA tumorigenesis and metastasis *in vivo*

**Figure 6:**
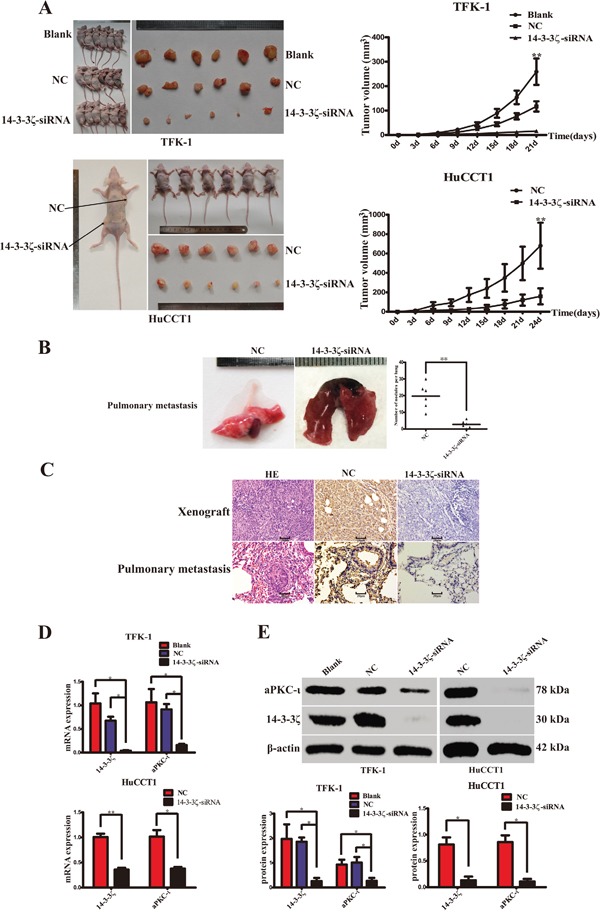
Silence of 14-3-3ζ suppress CCA cell proliferation, invasion and metastasis *in vivo* **A.** Tumor growth was evaluated in a nude mouse xenograft model after 21 days (TFK-1, upper panel) or 24 days (HuCCT1, lower panel) of treatment as indicated. Images were captured (left panels), and the growth curves were plotted (right panels). **B.** Tumor metastases to the lung were evaluated in the nude mouse pulmonary metastasis model after 6 weeks of treatment, as indicated. Representative image was captured (left panel). Statistical analysis was shown (right panel). **, *P*<0.001. **C.** Haematoxylin-eosin (HE; left panels) and 14-3-3ζ (middle and right panels) staining were used to illustrate the expression of 14-3-3ζ in the xenograft tumors (upper panel) and pulmonary metastasis tumors (lower panel) of CCA cells. Scale bar, 20μm. **D.** qRT-PCR was used to analyze 14-3-3ζ and aPKC-ι expression in the xenograft tumor (TFK-1, upper panel; HuCCT1, lower panel). **, *P*<0.001 and *, *P*<0.05. **E.** Western Blot was used to detect 14-3-3ζ and aPKC-ι protein expression in the xenograft tumor (TFK-1, left panel; HuCCT1, right panel). Representative image was captured (upper panel). Statistical analysis of the relative optical density of each band was shown (lower panel). Cells that were not transfected with virus were used as the blank control, and those that were transfected with an empty vector were used as the negative control (NC). β-actin was used as the loading control. *P<0.05.

*In vivo*, a subcutaneous tumor model and a pulmonary metastasis tumor model were established in nude mice to examine the therapeutic effects of 14-3-3ζ-siRNA in CCA (Figure 6A & 6B). The volumes of tumors arising from xenografts of 14-3-3ζ-siRNA-transfected CCA cells were smaller than those in the blank or negative control groups (TFK-1: *P*=0.001; HuCCT1: *P*<0.001; Figure 6A). And the tumors with invasive growth, irregular tumor borders and microsatellite lesions were more aggressive and more frequently presented in the untreated groups. Moreover, fewer pulmonary metastatic tumors were detected in the siRNA treatment groups (*P*<0.001; Figure 6B). IHC, qRT-PCR and WB were employed to further examine the expression of 14-3-3ζ and aPKC-ι in subcutaneous tumors or pulmonary metastasis tumors. Both at gene and protein levels, the expression of 14-3-3ζ and aPKC-ι were higher in the untreated groups than in the treatment groups (*P*<0.05; Figure 6C, 6D, & 6E). The assays showed that silencing of 14-3-3ζ significantly inhibited tumor growth, invasion and metastasis of CCA cells *in vivo*.

### 14-3-3ζ and aPKC-ι synergistically promote Snail-induced EMT via phosphorylation of GSK-3β

**Figure 7:**
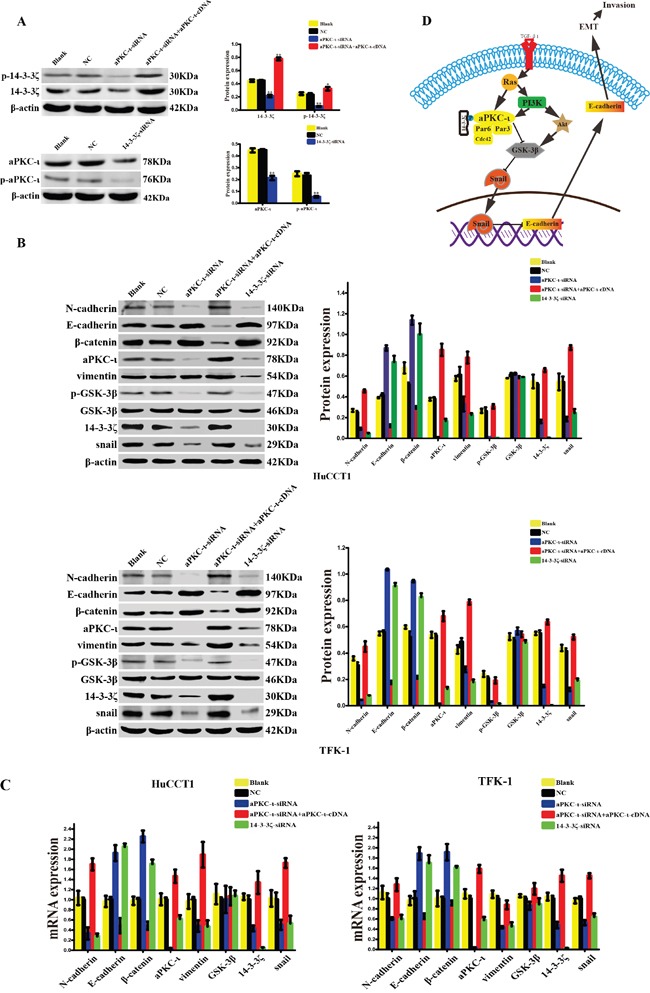
14-3-3ζ and aPKC-ι synergistically facilitate EMT of cholangiocarcinoma via GSK-3β/Snail signaling pathway **A.** The expression levels of 14-3-3ζ, p-14-3-3ζ (upper panel), and aPKC-ι, p-aPKC-ι (lower panel) in various cells treated with small interfering RNAs were determined by western blot. Representative image was shown (left panel). Statistical analysis was shown (right panel). Cells that were not transfected with virus were used as the blank control, and those that were transfected with an empty vector were used as the negative control (NC). β-actin was used as the loading control. **, *P*<0.001 and *, *P*<0.05. **B.** The protein expression levels of EMT-related markers, GSK3β, p- GSK3β and Snail in HuCCT1(upper panel) and TFK-1 cell(lower panel) treated with small interfering RNAs were determined by western blot. Representative image was shown (left panel). Statistical analysis was shown (right panel). Cells that were not transfected with virus were used as the blank control, and those that were transfected with an empty vector were used as the negative control (NC). β-actin was used as the loading control. **, *P*<0.001 and *, *P*<0.05. **C.** The gene expression levels of EMT-related markers, GSK3β, and Snail in HuCCT1(left panel) and TFK-1 cell (right panel) treated with small interfering RNAs were determined by qRT-PCR. Cells that were not transfected with virus were used as the blank control, and those that were transfected with an empty vector were used as the negative control (NC). β-actin was used as the loading control. **, *P*<0.001 and *, *P*<0.05. D. Schematic illustrations of the role of 14-3-3ζ and aPKC-ι in the EMT of CCA investigated in this study. 14-3-3ζ bind to and activate aPKC-ι through phosphorylation, and activation of aPKC-ι lead to the suppression of GSK-3β through phosphorylation, and furthermore, inhibition of GSK-3β results in the upregulation of Snail and downregulation of E-cadherin.

For the molecular mechanism of EMT, we further investigated the expression of phosphorylated 14-3-3ζ (p-14-3-3ζ) and phosphorylated aPKC-ι (p-aPKC-ι) which are the activated forms. Silencing of 14-3-3ζ decreased the expression of aPKC-ι and p-aPKC-ι. Similarly, the expression of p-14-3-3ζ was also decreased by targeted silencing of aPKC-ι, and was raised again in the aPKC-ι-siRNA rescue experiment (*P*<0.05; Figure 7A). Subsequently, we examined the expression of Snail (as the most important nuclear transcription factor in EMT) and GSK-3β (one of the upstream regulatory molecules of Snail) by WB and qRT-PCR. Silencing of 14-3-3ζ or aPKC-ι by siRNA, the markers of epithelial cell phenotype (E-cadherin and β-catenin) increased at the gene and protein levels in contrast to the markers of mesenchymal cell phenotype (N-cadherin and vimentin) decreased (*P*<0.05). Moreover, the expression of Snail and the phosphorylated GSK-3β (p-GSK-3β) was significantly decreased (*P*<0.05), the expression of GSK-3β did not change significantly (*P*>0.05). And in the aPKC-ι-siRNA rescue experiment, the expression of Snail and p-GSK-3β were raised again (*P*<0.05) (Figure 7B & 7C).

The above data demonstrated that the interaction between 14-3-3ζ and aPKC-ι promote the activated form of them by phosphorylation, and activation of aPKC-ι lead to activate the GSK-3β/Snail signaling pathway by p-GSK-3β, and promotes the EMT ultimately in CCA cells (Figure 7D).

## DISCUSSION

Cholangiocarcinoma (CCA) represents one of the most life-threatening tumor entities [1], and the main cause of death in patients is the invasion and metastasis of cancer [5]. In the early stages of the metastasis cascade, EMT is a critical process [7]. However, while substantial progress in the understanding of the molecular mechanisms of cancer has been made, we still lack sufficient insight into the mechanisms of EMT in patients with CCA. EMT can be induced by various signaling pathways and regulatory networks that, in many cases, overlap with developmental processes that are hijacked by cancer cells [26]. As a central hub in EMT, aPKC-ι is essential for the migration and invasion of multiple cancer cell lines and contributes to TGF-β-induced EMT [13]. Our previous study suggested that aPKC-ι is also involved in the EMT in CCA cells [14]. Meanwhile, 14-3-3ζ is ubiquitous in all eukaryocyte and acts as a central hub regulating many pathways involved in cellular transformation common to various cancers [18]. Over expression of 14-3-3ζ can increase the potential for invasion and metastasis by facilitating EMT [21, 23]. Nonetheless, the reports on the association of 14-3-3ζ with aPKC-ι in CCA are scarce.

Based on our previous studies, we enrolled the 64 consecutive patients with CCA, and further detected the co-expression of 14-3-3ζ, aPKC-ι and E-cadherin by IHC, qRT-PCR and WB. The results revealed that in contrast to E-cadherin low expression, 14-3-3ζ and aPKC-ι were co-overexpression in CCA tissues (Figure 1A, 1B & 1C) and there was a positive correlation between them (γ=0.406, *P*=0.001). Moreover, the χ^2^ analysis indicated that overexpression of 14-3-3ζ and aPKC-ι was significantly associated with poor tumor differentiation (14-3-3ζ: χ^2^=9.722, *P*=0.002; aPKC-ι: χ^2^=9.329, *P*=0.002) and advanced CCA TNM stage (14-3-3ζ: χ^2^=10.136, *P*=0.001; aPKC-ι: χ^2^=13.856, *P*<0.001) (Table 1). Therefor, CCA patients with high 14-3-3ζ or aPKC-ι expression had worse prognoses compared with those counterparts with low 14-3-3ζ or aPKC-ι expression (14-3-3ζ: χ^2^ =10.140, *P*= 0.001; aPKC-ι: χ^2^ =12.575, *P*<0.001, respectively) (Figure 1D). The multivariate analysis showed that the expression of 14-3-3ζ and aPKC-ι was an independent and significant risk factor for survival after curative resection (*P*=0.026; *P*=0.025, respectively) (Table 2). These clinical observations strongly suggested that 14-3-3ζ and aPKC-ι were oncogenes in CCA, and 14-3-3ζ expression was coupled with aPKC-ι expression to achieve a synergistic effect, and they may serve as useful prognostic biomarkers. Notably particularly, we found that superabundance of 14-3-3ζ and aPKC-ι were associated with a deficiency of E-cadherin in 64 CCA tissue samples(Figure 1A, 1B & 1C). These results indicated that 14-3-3ζ and aPKC-ι synergistically facilitate the progress of CCA via EMT.

This idea was further supported by protein and gene expression analyses, including CO-IP and step-by-step interference *in vitro*. CO-IP assay revealed that 14-3-3ζ could bind to aPKC-ι in CCA (Figure 2A). Furthermore, 14-3-3ζ or aPKC-ι knockdown with siRNA was associated with correspondingly down-regulated expression of both aPKC-ι and 14-3-3ζ (*P*<0.05) (Figure 2B&2C and Figure 3). And then, in aPKC-ι-siRNA rescue experiment, the expression of 14-3-3ζ was up-regulated accompanying with elevated aPKC-ι (*P*<0.05) (Figure 2B&2C and Figure 3). Our studies recommended that 14-3-3ζ forms a complex with aPKC-ι, and they regulate each other in CCA cells. 14-3-3ζ is a dimeric protein and forms a flattened horseshoe structure containing two grooves that can modulate binding to phosphorylated serine/threonine motifs on its target proteins [27]. Dimerization and target binding are dependent on the phosphorylation of 14-3-3ζ. Two prototype 14-3-3ζ binding motifs are RSXpSXP and RXY/FXXpSXP, where pS represents phosphoserine and X any amino acid [28, 29]. Three phosphorylation sites on 14-3-3ζ (Ser58, Ser184 and Thr232) have been identified. 14-3-3ζ regulates its target proteins through a number of mechanisms including: changing the conformation of the protein, affecting protein activity or stability, facilitating protein complex formation, or altering protein subcellular localization [18]. Phosphorylation of 14-3-3ζ at Ser184 residue converts 14-3-3ζ to 14-3-3σ, and enhances its interaction with PKC *in vitro*[30]. aPKC-ι is also a serine/threonine kinase, and the N-terminal of aPKC-ι contains a conserved regulatory region-PB1 (Phox-Bem1) area which is rich in cysteine sequence [31, 32]. aPKC-ι can be activated through specific protein-protein interactions mediated by a Phox-Bem1 (PB1) domain within the N-terminal regulatory domain [33]. Therefore, the PB1 area plays a crucial role in aPKC-ι promoting cell polarization, proliferation, survival, transformation and other biological activities [34, 35]. The phosphorylation sites include Ser217, Ser237/Ser238, and Ser35/Ser37 [36]. In the further study, we investigated the activated forms p-14-3-3ζ and p-aPKC-ι. Similarly, both p-14-3-3ζ and p-aPKC-ι expression were inhibited by siRNA, and p-14-3-3ζ expression was recovered in the aPKC-ι-siRNA rescue experiment (*P*<0.05) (Figure 7A). In combination of our research, we hypothesized that 14-3-3ζ bind to the PB1 area of aPKC-ι through phosphorylation site, and activate the functions of aPKC-ι. However, the specific phosphorylation sites were still under further study.

Meanwhile, our data provided a novel concept that a positive regulatory loop exists between 14-3-3ζ and aPKC-ι. However, it is worth to note that the loop remains largely unknown. The potential mechanisms could include: (1) Phosphorylation of 14-3-3ζ at Ser184 residue converts 14-3-3ζ to 14-3-3σ, and enhances its interaction with aPKC-ι *in vitro*[30]. (2) 14-3-3ζ protein forms stable homolo- or hetero-dimers structure, and feedback regulates 14-3-3ζ dimerization or expression alter the balance of 14-3-3ζ dimers [18]. (3) aPKC-ι activates MAPK signaling pathway, and mitogen-activated protein kinase-activated protein kinase 2 (MAPAKPK2) phosphorylates the Ser58 residue on 14-3-3ζ to disrupt binding by altering 14-3-3ζ dimerization [37]. (4) 14-3-3ζ is post-transcriptionally regulated by miR-375, leading to down regulation of both 14-3-3ζ mRNA and protein [38]; (5) The sub-cellular localization of 14-3-3ζ directs or limits interacting protein [39].

Activated 14-3-3ζ and aPKC-ι synergistically facilitate the progress of CCA via EMT. For insight into the molecular regulation of EMT, the TGF-β1-induced EMT model was successfully established and the changes in EMT markers were consistent with those described in the literature (Figure 4) [40]. Subsequently, silencing of 14-3-3ζ or aPKC-ι prevented alterations in the EMT markers from occurring after TGF-β induction (*P*>0.05) (Figure 4). Moreover, Snail, as the most important nuclear transcription factor in EMT, was decreased (*P*<0.05). Meanwhile, p-GSK-3β, one of the upstream regulatory molecules of Snail, was significantly decreased (*P*<0.05) (Figure 7B & 7C). However, in an aPKC-ι-siRNA rescue experiment, the conversion of EMT markers was recovered (*P*<0.05) (Figure 4), and both expression of Snail and p-GSK-3β were increased (*P*<0.05) (Figure 7B & 7C). A large number of studies have demonstrated that the nuclear transcription factor Snail inhibits the expression of E-cadherin [41] and is closely associated with cancer metastasis via EMT [42]. Furthermore, Snail is degraded by proteasome after GSK-3β-dependent phosphorylation [10]. The suppression of GSK-3β through phosphorylation of Ser 9 promotes Snail to be much more stable and reside exclusively in the nucleus to induce EMT. In fact, Snail and GSK-3β together function as a molecular switch for many signaling pathways that lead to EMT [10]. In summary, 14-3-3ζ binds to aPKC-ι and activate aPKC-ι through phosphorylation, and activation of aPKC-ι lead to the suppression of GSK-3β through phosphorylation of Ser 9, and furthermore, inhibition of GSK-3β results in the upregulation of Snail and downregulation of E-cadherin, and promotes the EMT ultimately in CCA cells (Figure 7D).

EMT endows malignant cells with migration and invasion [43]. So, invasion, migration and proliferation of CCA cells were investigated *in vitro* (Figure 5), and the subcutaneous tumor model and the pulmonary metastasis tumor model were established *in vivo* (Figure 6). After silencing of 14-3-3ζ significantly inhibited the tumor development, growth, invasion and metastasis of CCA cells. These data show that 14-3-3ζ promotes the EMT of CCA cells and that the silencing of 14-3-3ζ suppresses EMT-induced invasion and tumor metastasis. Therefore, directly targeting 14-3-3ζ may be a viable approach for anti-cancer therapy.

To sum up, our study demonstrates that 14-3-3ζ and aPKC-ι synergistically facilitate the progression of CCA and are independent factors on the survival prognosis. 14-3-3ζ binds to aPKC-ι through phosphorylation site, and activate the functions of aPKC-ι. Activation of aPKC-ι lead to the suppression of GSK-3β through phosphorylation, and inhibition of GSK-3β results in the upregulation of Snail and downregulation of E-cadherin, and promotes the EMT. Silencing of 14-3-3ζ suppresses EMT-induced invasion and metastasis. Our results suggest that 14-3-3ζ may be used as a prognostic biomarker and therapeutic target in patients with CCA.

## MATERIALS AND METHODS

### Patients and clinicopathological data

From January 2008 to June 2013, 64 consecutive patients underwent resection of cholangiocarcinoma at the Department of Biliary and Pancreatic Surgery, Affiliated Tongji Hospital, Tongji Medical College, Huazhong University of Science and Technology (HUST), Wuhan, China (Table S1 and S2). Ethical approval was obtained from the Affiliated Tongji Hospital Research Ethics Committee. From each subject, a fresh tumor sample and a matching sample from adjacent (2 cm away from tumor tissue) non-tumor tissue were collected and the detected by immunohistochemistry (IHC), qRT-PCR, and Western blotting. The cohort of 31 female and 33 male patients had a median age of 56.5 years (range 38~79 years). All patients had been confirmed by pathology, and had been followed up for at least 2 months or until death. The disease-free survival varied from 2 to 64 months (median 20 months). None of the patients had received any chemoradiotherapeutic agents in the pre-operation. Histopathologic diagnosis was based on the World Health Organization criteria [44], and the seventh edition of the tumor-node-metastasis (TNM) classification system was also used [45]. The tumors fell into two categories in terms of differentiation: well-differentiated (n=23), medium- or poor-differentiated (n=41). Meanwhile, TNM stages of tumors were as follows: 23 cases were in stage I, and 19 in stage II, and 18 in stage III, and 4 in stage IV, respectively. Besides, ten patients with choledochocyst served as normal controls.

### Cell lines, antibodies and reagents

Two human CCA cell lines, TFK-1— a extrahepatic bile duct carcinoma cell line [46] and HuCCT1 — a intrahepatic bile duct carcinoma cell line [47], were used in this study. The cell lines were kindly provided by professor Shengquan Zou (Department of Biliary and Pancreatic Surgery, Affiliated Tongji Hospital, Tongji Medical College, Huazhong University of Science and Technology, Wuhan, China). Professor Shenquan Zou purchased two cell lines from DSMZ (Braunschweig, Germany).

All the antibodies and reagents used in this study are detailed in Table S3 and S4.

### Immunohistochemistry (IHC) assay

The expression of aPKC-ι and 14-3-3ζ protein was immunohistochemically detected by streptavidin biotin compound (SABC) method as previously reported [48]. The same settings were used for each image obtained by using Nikon ECLIPSE C1 confocal microscope (Nikon Corporation, Japan). Immunohistochemical scores were assessed by 3 pathologists blind to patient conditions. Immunohistochemical staining of aPKC-ι, 14-3-3ζ and E-cadherin were regarded as positive when pale-yellow and buff-colored particles could be presented in the cell membrane and/or cytoplasm. Five different fields (40 × 10) for every section were observed randomly. The IHC results were semiquantitatively classified into the following five groups according to the staining percentage of positive cells: score 0, no positive cell; score 1, positive cells in <25% of the specimen; score 2, positive cells in 25%-50% of the specimen; score 3, positive cells in 50%-75% of the specimen; score 4, positive cells in >75% of the specimen [49]. Staining intensity was graded as follows: score 0, no staining; score1, pale yellow staining; score 2, buffy staining; score 3, strongly brown staining. The total score of each section was a cross-product by two parameters. Specimens staining were assessed as total score: negative (−), score 0; weakly positive (+), score 1-4; positive (++), score 5-8; powerful positive (+++), score 9-12. The total score: a score of ≤ 4 as low expression, and > 4 as high expression. Tumor, peritumoral, and normal tissues in every section were assessed separately.

### Cell culture and EMT model *in vitro*

TFK-1 and HuCCT1 cells were cultured in RPMI-1640 (HyClone, Beijing, China) containing 10% foetal bovine serum (FBS, Gibco, CA, USA), 100 U/ml penicillin and 100μg/ml streptomycin (Beijing Solarbio Science & Technology Co., Beijing, China) at 37°C in a 5% CO_2_ incubator [46]. Antibiotics were not added to the culture medium when cells were prepared for transfection with lentivirus transfection.

EMT was induced with TGF-β1 (PeproTech, New Jersey, USA). When expanded to 80~90% confluence, cells were treated with serum starvation in 0.2% FBS for 24h. And then, cells were incubated in serum starvation containing 10 ng/ml TGF-β1 for 5 days at 37°C and 5% CO_2_ incubator. Morphological changes were examined under a phase-contrast microscope (Model CKX41, Olympus, Tokyo, Japan).

### Lentivirus vector construction

Three recombinant lentivirus for 14-3-3ζ (Genbank access number: NM_001135699) and aPKC-ι (Genbank access number: NM_002740), which expressed 14-3-3ζ-specific siRNA, aPKC-ι-specific siRNA and aPKC-ι-specific cDNA respectively, were purchased from Shanghai Genechem Co., Ltd. (Shanghai, China). The Lentivector Expression System was composed of the vectors pGC-LV vector, pHelper 1.0 (gag/pol element) and pHelper 2.0 (VSVG element). The vector GV118 (U6–MC–Ubi–EGFP) stably expressed siRNA and a marker (GFP-RFP fusion protein) for 14-3-3ζ. Two vectors, GV112 (hU6-MCS-CMV-Puromycin) and GV341 (Ubi-MCS-3FLAG-SV40-Puromycin) stably expressed siRNA and cDNA respectively, and drug-resistant for aPKC-ι. The vectors pHelper1.0 and pHelper 2.0 contain virus package imperative elements.

The siRNA against the human 14-3-3ζ gene was designed by GeneChemas software package as: 14-3-3ζ-siRNA, 5′-CTGTGTTCTATTATGAGAT-3′, and was introduced into *Hpa*I/*Xho*I restriction sites of GV118. The siRNA against the human aPKC-ι gene was designed as: aPKC-ι-siRNA, 5′-TTTAGACTTTATGAGCTAA-3′, and was introduced into *Age*I/*Eco*RI restriction sites of GV112. The lentiviral vector hU 6-MCS-CMV-Puro^r^-negative containing the sequence 5′-TTCTCCGAACGTGTCACGT-3′, which had no significant homology to any known human or mouse genes, was used as a negative control. Besides, the cDNA of the human aPKC-ι gene, a fragment encoding the aPKC-ι sequence plus 1833 bp at both 5′- and 3′-flanking regions was amplified with the primers 5′-CCAACTTTGTGCCAACCGGTCGCCACCATGCCGACCCAGAGGGACAGCAGCACCATGTCCCACACGGTCGCAG-3′ (forward) and 5′-AATGCCAACTCTGAGCTTGACACATTCTTCTGCAGACATC-3′ (reverse) by PCR from human genomic DNA and then cloned into the AgeI/NheI sites of GV341. The lentiviral with empty vector was used as a negative control.

The recombinant viruses were packaged using the Lentivector Expression System. The lentiviral packaging and tittering were performed according to the manufacturer's protocol. The multiplicity of infection (MOI) of 14-3-3ζ-siRNA, aPKC-ι-siRNA, and aPKC-ι-cDNA all were 10.

### Virus transfection and establishment stable cell clones

The protocol of lentivirus infection was according to the GenePharma Recombinant Lentivirus Operation Manual (http://www.genepharma.com). TFK-1 or HuCCT1 cells had been placed at a density of 1×10^5^ in 6-well plate (Corning, NY, USA) for 24h. Then, cells had been infected with 2μl concentrated lentivirus in the presence of polybrebe (8μg/ml) for 72h. The cells with GFP-positive had been selected and amplified according to subculturing protocol at a ratio of 1:3 twice a week for 2 weeks to generate stable monoclonal cell lines. And the cells with puromycin-resistant gene had been selected for 2 weeks using puromycin (5μg/ml, Sigma-Aldrich, St. Louis, USA) to generate stable monoclonal cell lines. Besides, un-transfected TFK-1 and HuCCT1 served as blank controls. The expression of 14-3-3ζ and aPKC-ι in the aforementioned cell lines were confirmed by Immunofluorescence (IF), Quantitative Real-Time Polymerase Chain Reaction (qRT-PCR), and Western blot (WB) analysis.

### siRNA rescue experiment

The stable cells with aPKC-ι-siRNA were grown in 6-well plate, and had been re-infected with 2μl concentrated lentivirus, expressing aPKC-ι-cDNA, in the presence of polybrene (8μg/ml) for 72h. Then, the cells with puromycin-resistant gene were selected for 2 weeks by using puromycin (5μg/ml) to generate stable monoclonal cell lines. The lentivirus with empty vector was used as a negative controls and un-transfected cells served as blank controls. The expression of 14-3-3ζ, aPKC-ι and markers of EMT were detected by IF, qRT-PCR and WB.

### Total RNA extraction and quantitative real-time polymerase chain reaction (qRT-PCR)

Total RNAs were extracted from biliary duct or cells by the TRIzol (Life Technologies, California, USA). RNA concentrations of standards and specimens were determined by a UV-3000 spectrophotometer (Beckham, Germany). Subsequently, reverse transcription into cDNA was performed by using TransScript First-Strand cDNA Synthesis SuperMix kit (Bioer Serves Life Co., Ltd., Beijing, China) on LifePro Thermal Cycler (Hangzhou Bioer Technology Co., Ltd., Hangzhou, China). And then, the cDNA samples (2μl) were employed for qRT-PCR using TransStart™ Top Green qPCR SuperMix kit (Hangzhou Bioer Technology Co., Ltd., Hangzhou, China) to 40 cycles on the CFX Connect™ Real-Time System (Bio-Rad, Hercules, CA, USA). The primer sequences were provided by Biossci biotechnologies Company Limited (Wuhan, Hubei, China) (Table S5). All the assays were carried out according to manufacturers’ introductions [48]. The cycle time (Ct) values of the selected genes were first normalized with the value of β-actin of the same sample, and fold changes were calculated through relative quantification (2^−^^ΔΔCt^). The formula is 2^−^^ΔΔCt^ = 2^Control group (Ct value of Target gene - Ct value of β-actin)- experiment group (Ct value of Target gene - Ct value of β-actin)^, and all of the experiment were repeated 3 times.

### Total protein/phosphorylated protein extraction and western blotting WB

Total proteins were extracted from biliary duct tissues or cells using RIPA lysis buffer (Beyotime Institute of Biotechnology, Jiangsu, China) with 1 mM PMSF (Beyotime Institute of Biotechnology, Jiangsu, China) according to manufacturers’ introduction. The samples supplemented with phosphatase inhibitors (Sigma-Aldrich) for extraction of phosphorylated protein. After clarification (centrifugation at 10,000 × g for 15 min at 4°C), the soluble protein concentration was determined by the bicinchoninic acid (BCA, Beyotime Institute of Biotechnology, Jiangsu, China) method. For immunoblotting, 50μg total protein/phosphorylated protein samples had been incubated at 100°C for 5 min, separated by sodium dodecyl sulfate polyacrylamide gel electrophoresis (SDS-PAGE, Boster Biological Technology Co., Ltd., Wuhan, China) and electrotransferred onto a PVDF membrane (Amersham, Chalfont, UK). Immunoblotting was carried out as described previously [50]. Finally, enhanced chemiluminescence (ECL, Boster Biological Technology Co., Ltd., Wuhan, China) was used to visualize the samples on X-Omat S films (Amersham), and β-actin (1:2000, Beyotime Institute of Biotechnology, Jiangsu, China) was used as an internal control. To quantify the relative levels of protein expression, the intensity of the specific bands was estimated by the ImageJ2X analysis software package (National Institute of Mental Health, Bethesda, MD, USA). The multiple protein expression in experimental group compare with the control group, and the formula is (gray value of target protein - gray value of background) / (gray value of β-actin - gray value of background), and all of the experiment were repeated 3 times.

### Co-immunoprecipitation (CO-IP)

Total proteins were extracted and clarificated. A portion of the supernatant corresponding to 1 mg of total proteins had been pre-cleared for 2h at 4°C with rabbit antibody IgG (1μg, ProteinTech Group, Chicago, IL, USA) and protein A+G agarose beads (20μl, Beyotime Institute of Biotechnology, Jiangsu, China) to get rid of non- specific binding. Subsequently, the pre-cleared supernatant were incubated with 2μg of anti-aPKC-ι antibody (Cell Signaling Technology, MA, USA) or anti-14-3-3ζ antibody (Santa Cruz Biotechnology, Santa Cruz, CA, USA) overnight on a spinning wheel at 4°C, with a parallel containing 2μg rabbit antibody IgG as negative control, and a certain proportion of supernatant without any antibody (Input) was used as a positive control. Afterwards, 40μl of protein A+G agarose beads were dropwise added to bind to the antibodies for 3h at 4°C. The pellets were spun down at 2500rpm for 5 min, and washed 5 times with RIPA lysis buffer (1 ml) in sequence. The collected proteins were re-suspended in 20μl 1×SDS-PAGE loading buffer (Boster Biological Technology Co., Ltd., Ltd., Wuhan, China) and were instantaneous centrifuged with high speed to the bottom of the tubes. Following by boiling at 100°C for 5 min, the protein samples separated by SDS-PAGE and analyzed by WB using the primary antibody of anti-14-3-3ζ or anti-aPKC-ι, correspondingly. The intensity of the specific bands was estimated by Image J2X software package. The assays were repeated at least three times.

### Immunofluorescence (IF)

For IF analysis, the treatment cells had been incubated in 6-wells plate with cover-slips for 24h. After fixing in 4% paraformaldehyde (Google biological technology co., Ltd., Wuhan, China) for 15 minutes at room temperature, cells had been permeated in 0.4% Triton X-100 (Amresco, Ohio, USA) for 10 minutes. While non-specific binding had been removed by 1% BSA (Amresco, Ohio, USA) in 1× PBS (2 ml) for 30 minutes at 37°C, the cells were incubated with primary antibodies (14-3-3ζ, 1:25; aPKC-ι, 1:25; E-cadherin, 1:20) overnight at 4°C. Cys3-conjugated affinipure goat anti-rabbit IgG (H+L) (1:20, ProteinTech Group, Chicago, IL, USA) and fluorescein (FITC)-conjugated affinipure goat anti-rabbit IgG (H+L) (1:20, ProteinTech Group, Chicago, IL, USA) were used as secondary antibodies and incubated with cells for 1h at 37°C. Besides, nuclei were stained by DAPI (5μg/ml, Beyotime Institute of Biotechnology, Jiangsu, China) for 2 min at room temperature. Images were captured by utilizing a Carl Zeiss LSM710 laser scanning confocal microscope (Carl Zeiss, Oberkochen, Germany).

### Transwell cell invasion assay

For measuring the invasive ability of cells, the transwell chamber (8μm in pore size, Corning Incorporated, NY, USA) invasiveness assays were performed with 24-well transwell units. For invasion assays, the mixture of BD Matrigel (BD Biosciences, NJ, USA) and RPMI-1640 (90μl, 1:8) were pre-coated into the chamber inserts as the basilar membrane for overnight under sterile conditions. After hydrating the basilar membrane with 0.1% BSA solution, the treated cells were seeded into the upper chamber containing RPMI-1640 and 0.2% FBS albumin (250μl in total) at density of 1×10^5^ cell/chamber, and the 500μl medium containing 10% FBS was added into the lower chambers. The cells had been incubated for 48h. After the cells on top side of each insert being scraped off, the chambers were fixed in 4% paraformaldehyde and stained by 1% crystal violet (Sinopharm Chemical Reagent Co., Ltd., Shanghai, China) in sequence. Finally, the trans-well membrane was visualized under Nikon Digital ECLIPSE C1 system (Nikon Corporation). Photographs of 5 random fields across three replicate wells were captured for quantification analysis. Migrated cells were counted by the Image-Pro Plus v6.0 software (Media Cybernetics Inc.).

### Wound healing assay

The treated cells were seeded into 6-well plates at density of 2×10^5^ cells/well and incubated to 95% confluence. The monolayers of cells were scratched a line across by a 10μl pipette tip. Cell migration was recorded by phase contrast microscopy (Nikon Digital ECLIPSE C1 system, Nikon Corporation) at 48h after the scratch. Photographs of 5 random fields across three replicate wells were captured for quantification analysis. Migrated area was measured by the Image-Pro Plus v6.0 software (Media Cybemetics Inc., Bethesda, MD, USA).

### Colony formation assay

The treated cells were seeded into 6-well plates at density of 500 cells/well and were cultured in RPMI-1640 containing 20% fetal bovine serum, 100U/ml penicillin and 100μg/ml streptomycin at 37°C and 5% CO_2_ incubator. At day 14, the plates were fixed in 4% paraformaldehyde and stained by 1% crystal violet in sequence. Subsequently, the colonies with greater than 100μm in diameter were counted and analyzed by Alpha Innotech Imaging system (Alphatron Asia Pte Ltd, Singapore, Singapore).

### Tumorigenicity and metastasis assays *in vivo*

For *in vivo* research, 4 to 6-week-old female BALB/c (*nu/nu*) mice were housed under specific pathogen-free (SPF) conditions and cared for according to the institutional guidelines for animal care. Each experiments *in vivo* were performed with six mice in each group. All the animal experiments were performed in strict accordance with the Guidelines of the National Institutes of Health guidelines (NIH publication 86-23 revised 1985) and were approved by the Committee on the Ethics of Animal Experiments of the Tongji Medical College, HUST, Wuhan, China.

For tumorigenesis assay, TFK-1 cells with different level expression of 14-3-3ζ were subcutaneously injected into the upper right flank of nude mice, while TFK-1 cells untransfected with 14-3-3ζ-siRNA were used as a blank control, and transfected empty vector were saved as a negative control. HuCCT1 cells with 14-3-3ζ-siRNA were subcutaneously injected into the lower left flank of nude mice, while HuCCT1 cells containing empty vector were injected into the upper right flank of the same mice as a negative control. 2×10^6^ tumor cells were suspended in 100μl serum-free RPMI-1640 and injected subcutaneously. All mice were monitored once every 3 days and were sacrificed 3 weeks later. Tumor length and width were determined by a digital caliper. And tumor volume was calculated according to the following equation: V (volume, mm^3^) = 0.5× L (length, mm) × W^2^ (width, mm^2^). For groups in which the incidences of tumor were 100%, growth curves for tumors were plotted based on measured tumor volume within each experimental group at the indicated time points. After the xenograft tumors were resected, weighted and dissected, part of tumor tissues were prepared for immunostaining, and the remaining tumor tissues were used for qRT-PCR or WB assays. Two independent experiments were performed, and they gave similar results.

Pulmonary metastasis tumor model was employed to assess the effect of 14-3-3ζ on tumor metastasis. 2×10^6^ treated HuCCT1 cells were suspended in 100μl serum-free RPMI-1640 and injected via the tail vein of nude mice, while those with empty vector were used as a negative control (NC). All mice were monitored once every 3 days, and sacrificed 6 weeks later. The metastases tumor in the lungs were examined by necropsy. The number of the metastatic nodules in histologic sections from the midportion of each sample was used to evaluate tumor metastasis. Part of lung tissues were examined by IHC.

### Statistical analysis

Data were statistically analyzed by using the software package SPSS (version 19.0) for Windows supplied by the Statistics Teaching Room of Tongji Medical Collage, HUST. The results were presented as the mean ± SD (standard deviation) if data were quantitative nature. Statistical significance was assessed by two-tailed Student's t-test, analysis of variance (ANOVA) or Pearson's correlation test when applicable. Categorical data were analyzed by χ^2^ test. Kaplan-Meier and log-rank analysis was used to assess the survival between subgroups. A Cox proportional hazards model was used to determine the independent factors of survival based on the variables selected in univariate and multivariate analyses. A value of *P*<0.05 was considered statistically significant.

## SUPPLEMENTARY TABLES




