# Supplementary material for: 14-3-3ζ and aPKC-ι synergistically facilitate epithelial-mesenchymal transition of cholangiocarcinoma via GSK-3β/snail signaling pathway
Source: Oncotarget. 2016 Jul 8;7(34):55191–210. doi: 10.18632/oncotarget.10483 (PMC5342411; doi:10.18632/oncotarget.10483)
Supplement: Supplementary file 1 [file oncotarget-07-55191-s001.pdf]

14-3-3ζ and aPKC-ι synergistically facilitate epithelial-mesenchymal transition of cholangiocarcinoma via GSK-3β/snail signaling pathway

SUPPLEMENTARY TABLES

Supplementary Table S1: Clinical data of patients with cholangiocarcinoma

See Supplementary File 1

Supplementary Table S2: Clinicopathological characteristics of patients with CCA

Clinicopathological	n = 64	
**variables**	**number**	**percentage**
**Age (y)**		
≤ 60	39	60.94%
> 60	25	39.06%
**Gender**		
male	33	51.56%
female	31	48.44%
**Differentiation**		
well	23	35.94%
moderately/poorly	41	64.06%
**TNM stage**		
I - II	41	64.06%
III - IV	23	35.94%

Supplementary Table S3: Antibodies used in this study

Antigens	Manufacturers	Application
aPKC-ι	#5584, Cell Signaling Technology, Beverly, MA, USA	1:100 for IHC<br>1:250 for WB
aPKC-ι	sc-11399, Santa Cruz Biotechnology, Santa Cruz, CA, USA	1:25 for IF
14-3-3ζ	sc-1019, Santa Cruz Biotechnology, Santa Cruz, CA, USA	1:50 for IHC<br>1:500 for WB<br>1:25 for IF
E-Cadherin	20874-1-AP, ProteinTech Group, Chicago, IL, USA	1:50 for IHC<br>1:500 for WB<br>1:20 for IF
N-Cadherin	Ab51034, Abcam Inc., Cambridge, MA, USA	1:5000 for WB
Vimentin	10366-1-AP, ProteinTech Group, Chicago, IL, USA	1:500 for WB
β-Catenin	#1499, Cell Signaling Technology, Beverly, MA, USA	1:500 for WB
β-actin	AA128, Beyotime Institute of Biotechnology, Jiangsu, China	1:2000 for WB
Goat anti-rabbit IgG(H+L)	SA00001-2, ProteinTech Group, Chicago, IL, USA	1:3000 for WB, IHC
Goat anti-mouse IgG(H+L)	SA00001-1, ProteinTech Group, Chicago, IL, USA	1:3000 for WB
Goat anti-rabbit IgG(H+L) Cy3	SA00009-2, ProteinTech Group, Chicago, IL, USA	1:20 for IF
Goat anti-rabbit IgG(H+L) FICT	SA00003-2, ProteinTech Group, Chicago, IL, USA	1:20 for IF
Snail	#3879, Cell Signaling Technology, Beverly, MA, USA	1:500 for WB
GSK-3β	22104-1-AP, ProteinTech Group, Chicago, IL, USA	1:500 for WB
p- GSK-3β	sc-11757, Santa Cruz Biotechnology, Santa Cruz, CA, USA	1:500 for WB
p-14-3-3ζ	sc-101623, Santa Cruz Biotechnology, Santa Cruz, CA, USA	1:500 for WB
p-aPKC-ι	ab5813, Abcam Inc., Cambridge, MA, USA	1:500 for WB

Supplementary Table S4: Reagents used in this study

Reagent	Catalog NO.	Manufacturers
RMPI 1640	SH30809.01B	HyClone, Thermo Scientific Biochemical Products Co., Ltd., Beijing, China
Trypsin EDT	25200-056	Gibco, Life Technologies, Carlsbad, CA, USA
Fetal bovine serum(FBS)	16000-044	Gibco, Life Technologies, Carlsbad, CA, USA
Penicillin Streptomycin	P1400	Beijing Solarbio Scinece & Technology Co., Ltd., Beijing, China
Puromycin	PP2372-1KT	Sigma-Aldrich, St. Louis, USA
Recombinant human TGF-β1	#100-21	PeproTech, New Jersey, USA
DAPI staining solution	C1005	Beyotime Institute of Biotechnology, Jiangsu, China
Superstar ECL plus ready-to-use	AR1171	Boster Biological Technology Co., Ltd., Wuhan, China
TRIzol reagent	15596-026	Life Technologies, Carlsbad, California, USA
RIPA Lysis Buffer	P0013B	Beyotime Institute of Biotechnology, Jiangu, China
PMSF (100 mM)	ST506	Beyotime Institute of Biotechnology, Jiangsu, China
SABC kit	SA1022	Boster Biological Technology Co., Ltd., Wuhan, China
Protein A+G Agarose	P2012	Beyotime Institute of Biotechnology, Jiangsu, China
TransScript First-Strand cDNA Synthesis SuperMix kit	AT301	Bioer Serves Life Co., Ltd., Beijing, China
TransStart™ Top Green qPCR SuperMix kit	AQ131	Bioer Serves Life Co., Ltd., Beijing, China
BD Matrigel™ Matrix	356234	BD Biosciences, New Jersey, USA
SDS-PAGE loading buffer 5×	AR1112	Boster Biological Technology Co., Ltd., Wuhan, China
SDS-PAGE	AR0138	Boster Biological Technology Co., Ltd., Wuhan, China
4% paraformaldehyde	G1101	Google biological technology co., Ltd., Wuhan, China
TritonX-100	0694	Amresco, Ohio, USA
Tween 20	0777	Amresco, Ohio, USA
BSA powder	M231-22G-5PK	Amresco, Ohio, USA
Phosphatase Inhibitor Cocktail 1	P2850	Sigma-Aldrich, St. Louis, USA

Supplementary Table S5: Sequences of gene-specific primers used for qRT-PCR

Gene	Forward(5'-3')	Reverse(5'-3')
14-3-3ζ	TAGGTCATCTTGGAGGGTCG	GCTTCTTGGTATGCTTGTTGTG
aPKC-ι	TACGGCCAGGAGATACAACC	CATCTGGAGTGAGCTGGACA
E-Cadherin	CTGGACAGGGAGGATTTTGA	ACCTGAGGCTTTGGATTCCT
N-Cadherin	CGTGAAGGTTTGCCAGTGT	GCACAAGGATAAGCAGGATGA
Vimentin	AGAGAACTTTGCCGTTGAAGC	ACGAAGGTGACGAGCCATT
β-catenin	TGGTGACAGGGAAGACATCA	CCATAGTGAAGGCGAACTGC
β-actin	CTCTTCCAGCCTTCCTTCCT	ATGCTATCACCTCCCCTGTG
Snail	TCGGAAGCCTAACTACAGCGA	AGATGAGCATTGGCAGCGAG
GSK-3β	TGGAATCTGCCATCGGGATA	ATTGGGTTCTCCTCGGACCA
