# Supplementary material for: 14-3-3ζ and aPKC-ι synergistically facilitate epithelial-mesenchymal transition of cholangiocarcinoma via GSK-3β/snail signaling pathway
Source: Oncotarget. 2016 Jul 8;7(34):55191–210. doi: 10.18632/oncotarget.10483 (PMC5342411; doi:10.18632/oncotarget.10483)
Supplement: Supplementary file 2 [file oncotarget-07-55191-s002.docx]

**Table S1. Clinical data of patients with cholangiocarcinoma**

| **No.** | **No.of Inpatient** | **No.of Pathology** | **Gender** | **Age** | **Pathological diagnosis** | **Differentiation** | **TNM Stage** | **Survival time** |
| --- | --- | --- | --- | --- | --- | --- | --- | --- |
| CC1 | 1043816 | Ⅱ08-1767 | M | 67 | Well-differentiated adenocarcinoma invading the whole layer. | Well | II | 49 months |
| CC2 | 1045497 | Ⅱ08-2454 | F | 45 | Medium/Poorly differentiated adenocarcinoma invading the whole layer of bile duct with hilar and duodenum lymph node metastasis. | Medium/Poorly | III | 19 months |
| CC3 | 1046916 | Ⅱ08-3364 | F | 79 | Well-differentiated adenocarcinoma invading the whole layer of cystic duct of gallbladder. | Well | II | 12 months |
| CC4 | 1053741 | Ⅱ08-07356 | F | 42 | Poorly differentiated adenocarcinoma, immunohistochemistry: PCK (+), CK20 (+), the expression of LCA is dissatisfied, CK19 (-), hepatocyte (-). | Poorly | III | 16 months |
| CC5 | 1058881 | Ⅱ08-10892 | M | 66 | Moderately differentiated adenocarcinoma invading the whole layer of common bile duct. | Medium | II | 43 months |
| CC6 | 1063111 | Ⅱ08-14549 | F | 67 | Poorly differentiated adenocarcinoma invading the whole layer of common bile duct | Poorly | I | 34 months |
| CC7 | 851620 | Ⅱ08-18164 | M | 66 | Well differentiated adenocarcinoma. | Well | I | >60 months |
| CC8 | 1043901 | Ⅱ08-1969 | M | 34 | Moderately differentiated adenocarcinoma invading the whole layer of common bile duct. | Medium | I | >60 months |
| CC9 | 1072667 | Ⅱ08-19558 | F | 43 | Moderately differentiated adenocarcinoma with bile duct tumor thrombus. | Medium | IV | 14 months |
| CC10 | 1073272 | Ⅱ08-20509 | M | 55 | Well differentiated adenocarcinoma (hilar bile duct) invading the whole layer of bile duct and sero muscular layer of gallbladder. | Well | III | 31 months |
| CC11 | 1073926 | Ⅱ08-21575 | M | 70 | Well differentiated adenocarcinoma with bile duct tumor thrombus. | Well | IV | 4 months |
| CC12 | 1077679 | Ⅱ08-23779 | M | 40 | Well differentiated papillary adenocarcinoma invading the gallbladder | Well | II | >60 months |
| CC13 | 1077829 | Ⅱ08-23210 | M | 61 | Medium/Poorly differentiated adenocarcinoma invading the whole layer of bile duct. | Medium/Poorly | II | 23 months |
| CC14 | 1082285 | Ⅱ08-33338 | F | 47 | Well differentiated adenocarcinoma invading the mucosa and muscular of duodenal. | Well | III | >60 months |
| CC15 | 1090976 | Ⅱ08-29656 | M | 69 | Medium differentiated adenocarcinoma invading the whole layer of bile duct. | Medium | II | 16 months |
| CC16 | 1334416 | Ⅱ08-30075 | M | 61 | Medium/Poorly differentiated adenocarcinoma, immunohistochemistry: CK7 (+), CK19 (+), EMA (+), CK20 (scattered in little +), CKX-2 (+), Villin (+), Hepatocyte (-), Glypican-3 (-), Ki-67 LI 50-60%. | Medium/Poorly | III | 15 months |
| CC17 | 1100607 | Ⅱ09-01106 | F | 62 | Well differentiated adenocarcinoma. | Well | I | >60 months |
| CC18 | 1104491 | Ⅱ09-03540 | F | 75 | Well differentiated adenocarcinoma. | Well | I | 31 months |
| CC19 | 1108751 | Ⅱ09-05743 | F | 66 | Medium differentiated adenocarcinoma invading the whole layer of bile duct and the bottom of the gallbladder. | Medium | III | 12 months |
| CC20 | 1119817 | Ⅱ09-14156 | M | 57 | Well differentiated adenocarcinoma invading the liver and the lateral wall of cystic duct of gallbladder. | Well | III | >60 months |
| CC21 | 1132639 | Ⅱ09-23760 | M | 44 | Poorly differentiated adenocarcinoma. | Poorly | I | 43 months |
| CC22 | 1163548 | Ⅱ10-03211 | M | 68 | Medium differentiated adenocarcinoma. | Medium | I | 12 months |
| CC23 | 1174742 | Ⅱ10-09319 | M | 55 | Medium/Poorly differentiated adenocarcinoma. | Medium/Poorly | I | 40 months |
| CC24 | 1192893 | Ⅱ10-22071 | M | 61 | Well differentiated adenocarcinoma invading the whole layer of bile duct and liver | Well | II | >48 months |
| CC25 | 1193353 | Ⅱ10-22353 | F | 50 | Medium/Poorly differentiated adenocarcinoma, Immunohistochemistry: CK19 (+), CK8 / 18 (+), EMA (+), Hepatocyte (-) | Medium/Poorly | III | 2 months |
| CC26 | 1193474 | Ⅱ10-22657 | F | 55 | Well differentiated adenocarcinoma invading the left lobe and caudate lobe of liver. | Well | III | 13 months |
| CC27 | 1198495 | Ⅱ10-25550 | F | 61 | Medium differentiated adenocarcinoma invading the whole layer of bile duct and the bottom of the gallbladder. | Medium | III | 10 months |
| CC28 | 1203918 | Ⅱ10-29100 | M | 56 | Medium differentiated adenocarcinoma. | Medium | I | 34 months |
| CC29 | 1206023 | Ⅱ10-30668 | M | 43 | Medium differentiated adenocarcinoma invading the whole layer of bile duct. | Medium | II | 22 months |
| CC30 | 1214169 | Ⅱ10-35092 | M | 64 | Medium differentiated adenocarcinoma. | Medium | I | 28 months |
| CC31 | 1219238 | Ⅱ11-03010 | F | 60 | Well differentiated adenocarcinoma invading the whole layer of bile duct. | Well | II | >36 months |
| CC32 | 1228254 | Ⅱ11-04110 | M | 73 | Medium differentiated adenocarcinoma invading the liver. | Medium | III | 9 months |
| CC33 | 1233864 | Ⅱ11-08563 | F | 56 | Medium differentiated adenocarcinoma invading the whole layer of bile duct. | Medium | II | >36 months |
| CC34 | 1237428 | Ⅱ11-13473 | M | 54 | Medium differentiated adenocarcinoma. | Medium | I | 20 months |
| CC35 | 1237765 | Ⅱ11-11090 | F | 53 | Poorly differentiated adenocarcinoma invading the whole layer of bile duct. | Poorly | II | 17 months |
| CC36 | 1245590 | Ⅱ11-16793 | M | 48 | Medium/Poorly differentiated adenocarcinoma invading the whole layer of bile duct | Medium/Poorly | II | 16 months |
| CC37 | 1247366 | Ⅱ11-18693 | F | 78 | Medium differentiated adenocarcinoma. | Medium | I | 3 months |
| CC38 | 1250816 | Ⅱ11-20743 | F | 66 | Medium differentiated adenocarcinoma (hilar bile duct) invading the whole layer of bile duct. | Medium | II | 15 months |
| CC39 | 1252981 | Ⅱ11-22324 | M | 47 | Well differentiated adenocarcinoma. | Well | I | >36 months |
| CC40 | 1254397 | Ⅱ11-23223 | F | 60 | Medium differentiated adenocarcinoma invading the whole layer of bile duct. | Medium | II | 28 months |
| CC41 | 1254925 | Ⅱ11-23185 | M | 70 | Well differentiated adenocarcinoma. | Well | I | 22 months |
| CC42 | 1260387 | Ⅱ11-26775 | M | 57 | Well differentiated adenocarcinoma | Well | I | >36 months |
| CC43 | 12676524 | Ⅱ11-32018 | M | 71 | Medium differentiated adenocarcinoma. | Medium | I | 17 months |
| CC44 | 1272037 | Ⅱ11-35400 | M | 43 | Medium differentiated adenocarcinoma. | Medium | I | 33 months |
| CC45 | 1272858 | Ⅱ11-34688 | M | 64 | Well differentiated adenocarcinoma. | Well | I | >36 months |
| CC46 | 1275841 | Ⅱ11-39755 | F | 65 | Well differentiated adenocarcinoma invading the whole layer of bile duct. | Well | II | >36 months |
| CC47 | 1072667 | Ⅱ11-41161 | F | 60 | Medium differentiated adenocarcinoma (Intrahepatic cholangiocarcinoma). | Medium | I | 34 months |
| CC48 | 1278149 | Ⅱ11-42607 | M | 66 | Poorly differentiated adenocarcinoma, immunohistochemistry: the right branch of Portal vein wall: PCK (+), EMA (-), CK8/18 (+), CK19 (-);Bile duct of caudate lobe: PCK (-), EMA (-), CK8/18 (+), CK19 (+). | Poorly | III | 5 months |
| CC49 | 1001799055 | Ⅱ11-19450 | F | 49 | Medium/Poorly differentiated adenocarcinoma invading the cyst duct of gallbladder. | Medium/Poorly | III | 14 months |
| CC50 | 1313159 | Ⅱ12-19521 | F | 54 | Medium/Poorly differentiated adenocarcinoma, immunohistochemistry: CK19(+),Ki-67 LI:50%. | Medium/Poorly | III | 15 months |
| CC51 | 1317244 | Ⅱ12-23819 | F | 41 | Medium/Poorly differentiated adenocarcinoma invading the whole layer of bile duct and the sero muscular layer of gallbladder. | Medium/Poorly | III | 17 months |
| CC52 | 1001564748 | Ⅱ12-37729 | F | 47 | Medium/Poorly differentiated adenocarcinoma(hilar bile duct) | Medium | I | >24 months |
| CC53 | K000020787 | Ⅱ12-41912 | M | 49 | Medium/Poorly differentiated adenocarcinoma(hilar bile duct) | Medium | II | 21 months |
| CC54 | 1001259021 | Ⅱ12-43483 | M | 41 | Medium/Poorly differentiated adenocarcinoma invading the whole layer of bile duct and the wall around he pancreatic tissue with lymph node metastases ( lymph node around gallbladder 1/2, Group 8 1/1). | Medium | III | 9 months |
| CC55 | 1001669458 | Ⅱ12-48090 | F | 60 | Well differentiated adenocarcinoma. | Well | I | >24 months |
| CC56 | 1002288628 | Ⅱ12-11749 | F | 54 | Well differentiated adenocarcinoma胆总管下端高分化腺癌 | Well | I | >24 months |
| CC57 | 1001793200 | Ⅱ13-09166 | M | 51 | Medium differentiated adenocarcinoma with lymph node metastases ( group 13A 1/2, Group 14 1/1); Chronic cholecystitis with adenomyosis hyperplasia. | Medium | III | >12 months |
| CC58 | 1001905099 | Ⅱ13-13680 | F | 59 | Medium/Poorly differentiated adenocarcinoma (hilar bile duct)invading the whole layer of bile duct. | Medium | IV | >12 months |
| CC59 | 13100736061 | Ⅱ13-19412 | F | 63 | Well differentiated adenocarcinoma(Right intrahepatic bile duct and hilar bile duct). | Well | II | >12 months |
| CC60 | 1002268415 | Ⅱ13-40501 | F | 47 | Medium differentiated adenocarcinoma(Caudate lobe of liver) invading the right lobe of liver and gallbladder. | Medium | III | >12 months |
| CC61 | 1002568132 | Ⅱ13-30041 | F | 54 | Medium/Poorly differentiated adenocarcinoma (hilar bile duct) invading the nerves tissue of bile duct with lymph node metastases (6/8); Intrahepatic bile duct Epithelial Hyperplasia and focal fatty degeneration of liver cells. | Medium/Poorly | IV | 8 months |
| CC62 | 1218010 | Ⅱ13-36783 | M | 47 | Medium differentiated adenocarcinoma (Intrahepatic bile ducts). | Medium | I | >12 months |
| CC63 | 1246504 | Ⅱ13-40501 | F | 56 | Well differentiated adenocarcinoma（Duodenal ampulla）invading the whole layer of duodenum and adjacent tissue of pancreas. | Well | III | >12 months |
| CC64 | 1305157 | Ⅱ13-43466 | M | 38 | Well differentiated adenocarcinoma（Intrahepatic bile ducts of Segment Ⅵ）invading the wall of gallbladder. | Well | II | >12 months |
